# Opposing roles of prelimbic and infralimbic dopamine in conditioned cue and place preference

**DOI:** 10.1007/s00213-013-3414-0

**Published:** 2014-01-16

**Authors:** Anja Hayen, Saira Meese-Tamuri, Amy Gates, Rutsuko Ito

**Affiliations:** 1 Department of Experimental Psychology, University of Oxford, South Parks Road, Oxford, OX1 3UD UK; 2 Department of Psychology, University of Toronto Scarborough, 1265 Military Trail, Toronto, ON M1C 1A4 Canada

**Keywords:** Pavlovian learning, Medial prefrontal cortex, Amphetamine, Reward

## Abstract

**Rationale:**

Increasing evidence points to the prelimbic (PL) and infralimbic (IL) cortices of the medial prefrontal cortex (mPFC) and their dopaminergic innervations subserving opposing roles in the regulation of instrumental behavior. However, it is at present unclear if they hold similar roles in the regulation of Pavlovian learning.

**Objective:**

The present study investigated the role of the dopaminergic innervations of the PL and IL in the modulation of Pavlovian appetitive cue and place conditioning, previously shown to be dependent on the basolateral amygdala and hippocampus, respectively.

**Methods:**

Rats received preconditioning microinfusions of d-amphetamine, *cis*-flupenthixol, or vehicle solution directly into the PL or IL and were trained to simultaneously acquire conditioned cue and place preference in a radial maze.

**Results:**

Preconditioning blockade of dopamine neurotransmission in the PL and amphetamine microinfusions in the IL had the same effect of attenuating place conditioning. In contrast, place conditioning remained intact following preconditioning amphetamine microinfusions in the PL and dopamine receptor blockade in the IL. Instead, conditioned cue preference was attenuated following IL dopamine receptor blockade.

**Conclusion:**

These data indicate that PL dopaminergic mechanisms are critical for the acquisition of appetitive place learning, while IL dopamine may oppose the influence of PL dopamine upon hippocampal-dependent learning. Furthermore, they implicate a functional reciprocity between mPFC and associated subregions of the nucleus accumbens in the regulation of limbic information processing.

## Introduction

The medial prefrontal cortex (mPFC) is associated with a wide range of cognitive and mnemonic functions that include executive control, decision making, short-term and long-term memory, and regulation of instrumental learning and emotional behaviors. Its functional diversity is indicative of its adaptive importance in the prediction, optimization, and allocation of resources to changing task demands and rules (Euston et al. 2012). The prelimbic (PL) and infralimbic (IL) cortices are subdivisions of the rodent mPFC that are anatomically and functionally distinct, and accumulating evidence points to their opposing functions in the control of learned motivated behavior. More specifically, the IL cortex is implicated in the inhibitory control (extinction) of conditioned fear responses and conditioned drug-seeking behavior (Quirk and Mueller 2008) and the PL in *initiating* conditioned fear responses (Vidal-Gonzalez et al. 2006) and cocaine-seeking behavior (Peters et al. 2008). Furthermore, IL and PL cortices differentially mediate stimulus–response (habit) and action–outcome (goal-directed) learning, respectively (Coutureau and Killcross 2003), which are two forms of learning that can be thought to compete for control over instrumental responding and are highly prone to disruption in addiction (Everitt and Robbins 2005).

Pharmacological studies involving *post*-*training* manipulations of the mesocortical dopamine system point to the importance of dopaminergic mechanisms in the IL in regulating the balance of habit and action control over instrumental behavior (Hitchcott et al. 2007) and PL dopamine in the control of goal-directed responding (Naneix et al. 2009). Repeated systemic administration of amphetamine also facilitates the formation of habit, such that instrumental responding is no longer sensitive to outcome devaluation (Nelson and Killcross 2006; Nordquist et al. 2007), highlighting the importance of the mesocortical–limbic dopamine system, but in particular, the dopaminergic innervation of the dorsal striatum in the regulation of instrumental behavior (Faure et al. 2005). It is at present unclear whether the mesocortical dopamine system is involved in the modulation of learned motivated behavior at the acquisition stage.

The present study was designed to test the hypothesis that PL and IL dopamine (DA) neurotransmission is involved in the modulation of limbic influences upon appetitive Pavlovian conditioning *at the point of learning*. We previously demonstrated that limbic information mediated by the hippocampus (HPC, spatial information) and basolateral amygdala (BLA, discrete cue information) competes to gain control over Pavlovian reward learning and that repeated systemic amphetamine administration and repeated intra-accumbens shell (but not intra-core) amphetamine infusions cause dysregulation of this process, *enhancing* hippocampal control over Pavlovian approach behavior while attenuating BLA-dependent learning (Ito and Canseliet 2010; Ito and Hayen 2011). The IL and PL regions are integral components of the limbic-cortico-striatal circuitry and ideally positioned to regulate the balance of limbic (HPC vs. BLA) control over appetitive behavior. Both regions receive converging inputs that are mutually inhibitory from the HPC and BLA and a rich dopaminergic innervation from the ventral tegmental area (Hoover and Vertes 2007; Ishikawa and Nakamura 2003; Jay and Witter 1991). A further goal of the present study was to determine if dopamine manipulations in the IL and PL would mirror the effects seen following nucleus accumbens (NAc) core and shell dopamine manipulations, given their differential projection patterns to the NAc core and shell: the PL region projects extensively throughout the NAc shell and core and the IL projects more selectively to the NAc shell (Chiba et al. 2001; Vertes 2004).

## Materials and methods

### Subjects

Subjects were 50 male Lister hooded rats (Charles River Laboratories) weighing ∼330–400 g at the time of surgery. They were housed in groups of two or three in a room held at a constant temperature of 21 °C, under a 12-h light/dark cycle (lights on at 7:00 A.M.). Water was available ad libitum, but before the start of behavioral testing, food (laboratory chow, Purina) was restricted to 20 g of lab chow/day, sufficient to maintain preoperative/treatment body weight and growth. All experiments were conducted during the light phase and in accordance with the United Kingdom 1986 Animals (Scientific Procedures) Act Project License No. 30/2561.

### Surgery

All rats were anesthetized with isoflurane (Abbott Laboratories) and placed in a stereotaxic frame (Kopf) with the incisor bar set at −3.3 mm below the interaural line. A 26-gauge bilateral guide cannula (Plastics One) was then implanted, targeting the IL or the PL using the following coordinates (in millimeter from bregma): AP +2.6 (from bregma), *L* = ±0.75, and DV V = −3.90 (from SS at bregma) and AP = +3.0, *L* = ±0.6, and DV = −2.85, respectively. The guide cannula was anchored to the skull with dental cement (Kemdent Works) and skull screws. Stainless steel stylets (Plastics One) were placed in the guide cannulae to maintain their patency throughout the training period. Following surgery, rats were allowed a recovery period of at least 7 days before the commencement of behavioral testing, with food available ad libitum.

### Conditioned cue and place preference task

#### Radial arm maze apparatus

Behavioral testing took place in an automated six-arm radial maze (Med Associates) placed on a rotatable table elevated 80 cm from the floor. The maze consisted of six enclosed arms [45.7 cm (*L*) × 16.5 cm (*H*) × 9.0 cm (*W*)] emanating from a central hexagonal hub compartment with six automatic stainless steel guillotine doors allowing access to the arms. Arms were enclosed by Plexiglas walls and a removable Plexiglas lid and contained a grid floor. At the end of each arm was a receding well consisting of a stainless steel tray that could be connected up to a syringe pump for the delivery of sucrose solution (Med Associates). Each arm was also equipped with two sets of infrared beams located 2 and 3 cm away from the entrance of the arm to monitor an animal’s entry into and exit out of the arm.

The maze itself was placed in a testing room with various extramaze cues (stools, set of drawers, curtain), which remained in the same positions throughout the experiment. The maze was wiped down with ethanol solution after each session to eliminate odor traces, and the maze was randomly rotated left or right by varying degrees (60°, 120°, or 180°) at the end of the testing day to minimize conditioning to intramaze cues.

#### General microinfusion procedure

All rats were habituated to the infusion room and to gentle hand restraint for 5 min on each of the 3 days before the start of drug infusion. In addition, just before the first habituation session, each animal underwent a vehicle infusion (0.5 μl of saline) to minimize the mechanical effects of subsequent infusions, as well as to habituate the animals to the infusion procedure. For subsequent drug infusions, rats were held by hand while bilateral 33-gauge microinjectors projecting beyond the tip of the guide cannula by 1.5 mm were placed in the guide cannula. Drug infusions were conducted over 1 min using an infusion pump (Harvard Apparatus) mounted with a 5 μl Hamilton syringe, and the injector was left in place for a further 1 min to ensure diffusion of the drug away from the tip before removal.

All drug infusions were performed in a room separate from the animals’ housing and behavioral testing environment, following which the animals were transported to the testing room. Behavioral testing began 10–15 min after the end of the infusion.

### Experimental procedure

#### Habituation

Following a single vehicle infusion, all rats were given two 7-min habituation periods (one in the morning, one in the afternoon) on the day before the first drug infusion and conditioning session. They were initially placed in the central hub of the apparatus. After an adaptation time of 1 min in the hub, all six guillotine doors were opened and the rats were free to explore the whole maze for a further 6 min (with the floor insert cue placed in one of the arms). Two short sessions of habituation periods were given (rather than one 14-min session), as this enabled better acclimatization to the noise of the automated guillotine door opening and closing.

#### Drug microinfusions (sessions 1–7)

**Fig. 1:**
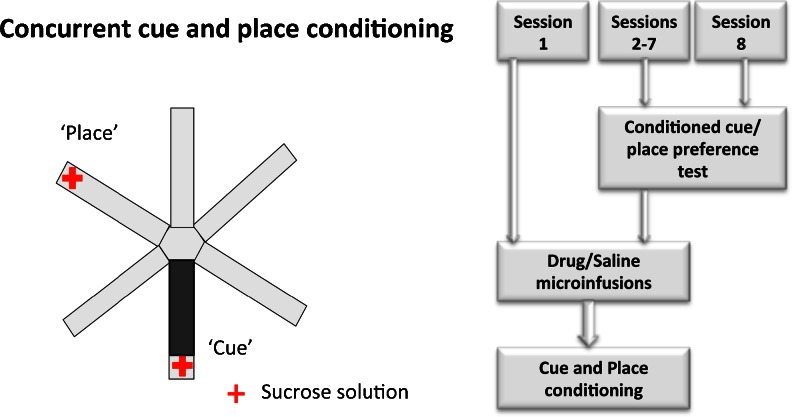
Schematic diagram (*left*) of the radial maze apparatus showing the availability of sucrose at the end of two arms (place and cue arms) in a conditioning session. The flow chart (*right*) shows the sequence of events in the concurrent cue and place conditioning paradigm. Following the habituation day, animals received seven daily conditioning sessions, each preceded by drug (amphetamine or flupenthixol) or saline infusions in a separate room. In addition, a conditioned cue/place preference test (5 min) was conducted in a drug-free state on sessions 2–8 (before the drug infusions) to monitor the rates of acquisition of cue and place conditioning

Rats were assigned to one of three infusion groups: amphetamine (*n* = 18), *cis*-flupenthixol (*n* = 18), or saline vehicle (*n* = 14). For each of the seven sessions, rats in the amphetamine infusion group received a bilateral infusion of 10 μg/0.5 μl d-amphetamine (Sigma-Aldrich) dissolved in sterile 0.9 % saline before the conditioning session. Similarly, rats in the flupenthixol group received a bilateral infusion of 20 μg/0.5 μl of dopamine D1/D2 receptor antagonist, *cis*-flupenthixol (Sigma-Aldrich), dissolved in sterile 0.9 % saline before each conditioning session. The drug doses were selected on the basis of pilot work indicating minimal effects on locomotor activity and anxiety levels, as well as previous studies demonstrating selective behavioral effects on PPI and expression of conditioned fear with these particular doses (Lacroix et al. 2000; Pezze et al. 2003). Animals in the saline vehicle group received a bilateral infusion of 0.5 μl of 0.9 % sterile saline solution. All drug infusions were spaced at least 24 h apart (see Fig. 1).

#### Concurrent cue and place conditioning (sessions 1–7)

Following each drug infusion, rats were brought into the behavioral testing room to start the conditioning session. Rats were initially placed in the central hub compartment of the radial maze for 30 s. They were then confined in each of the six arms for 2 min, with the order of presentation of the arms randomized across sessions for each animal. The rat received five aliquots of 0.3 ml of 20 % sucrose solution within the 2-min confinement period in two arms: (1) the “cue” arm, which contained a continuous black rubber floor insert (cue conditioning) that was moved between different arms in a pseudorandom fashion (all arms except the place arm) between sessions, and (2) the “place” arm, which was fixed in a particular spatial location (place conditioning). In summary, rats received sucrose reward in the arm with the floor insert (cue arm) regardless of its spatial location and in the arm that happened to occupy a fixed spatial location (place arm) in that particular session. The maze was rotated at the end of each day to minimize conditioning to the arms themselves or other intramaze cues. The rats did not receive any reward in the remaining four arms [non-rewarded (NR)].

#### Conditioned cue and place preference tests (sessions 2–8)

On the day after each conditioning session, rats were given a conditioned cue and place preference test in a drug-free state (before the next drug infusion and conditioning session) to assess the degree of conditioning to the cue and place. They were given 5 min to explore the entire apparatus, in the absence of any reward, and the time spent in each arm was recorded. A total of seven preference tests were conducted for each animal. The last test (test 7) in session 8 was not followed by a drug infusion or conditioning session.

### Histological procedure

All rats were anesthetized with sodium pentobarbitone (1.5 ml/animal, 200 mg/ml Euthatal, Rhone Merieux) and perfused intracardially via the ascending aorta with 0.01M PBS, followed by 10 % formalin saline. Brains were then removed, stored in 10 % formalin, and transferred to a 30 % sucrose cryoprotectant solution before sectioning. Coronal sections (50 μm) of the brain were cut using a freezing microtome and were then stained with cresyl violet, to be viewed under the microscope for the verification of cannula placements.

### Data analysis

All data were analyzed using the SPSS statistical package version 19.0 (SPSS Inc., Chicago, IL). Data generated for each test session (5 min) consisted of the absolute time spent in each of the six arms of the radial maze as well as the time spent in the hub. Based on previous findings (Ito and Hayen 2011) that the acquisition of conditioned place preference is an incremental process, and will only begin to be established after six to seven conditioning sessions in the control (saline) groups, a three-way repeated measures analysis of variance (ANOVA) was conducted on the raw data (time spent) obtained from the final conditioned cue and place preference test (test 7 in session 8) with region (cannula placement in prelimbic cortex or infralimbic cortex) and drug treatment (amphetamine, flupenthixol, and saline vehicle) as between-subjects factors and arm [cue, place, and non-rewarded (mean of time spent in the four non-rewarded arms)] as a within-subjects factor. Furthermore, in order to verify within-subject changes in the time spent in the cue and place arms before, and after the conditioning sessions, we compared the time spent in each arm in tests 1–7 (5 min) with the preconditioning baseline data generated in the first 5 min of the second 7-min habituation session. A four-way ANOVA was conducted on the change in the time spent in the respective arms for the tests, with drug treatment (amphetamine, flupenthixol, and vehicle) and region (prelimbic, infralimbic) as the between-subjects factors. Any significant three-way interactions were further explored using simple effects analyses. Subsequent post hoc comparisons for simple effects were performed with a Bonferroni correction.

## Results

### Cannulae placement

**Fig. 2:**
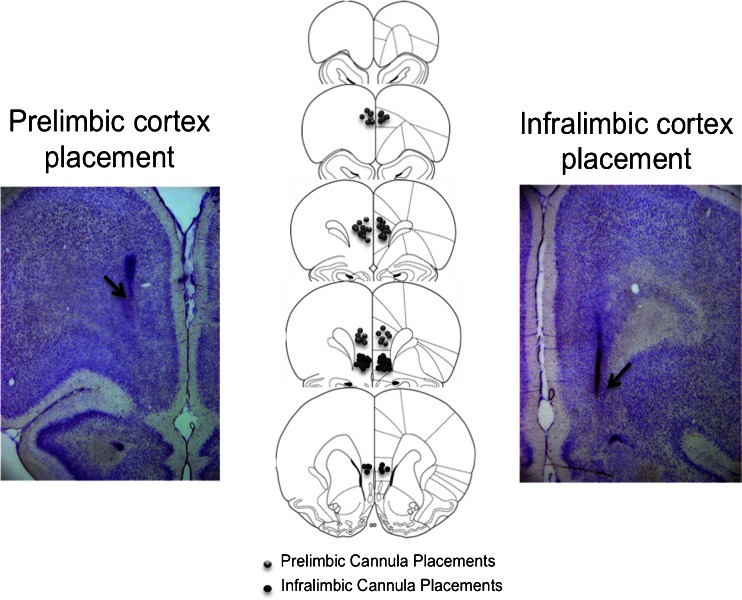
Schematic and photomicrographic representation of the locations of injector tips in the prelimbic cortex (*left*) and infralimbic cortex (*right*), based on Paxinos and Watson’s stereotaxic atlas of the rat brain (1997)

A schematic diagram and representative photomicrographs of the placement of injector tips within the prelimbic and infralimbic cortical regions are shown in Fig. 2, based on Paxinos and Watson’s stereotaxic atlas of the rat brain (1997). Data from four animals were excluded from statistical analyses in the prelimbic placement group due to incorrect placements that were too dorsal, and data from two animals were excluded from statistical analyses in the infralimbic placement group due to placements that were too ventral. The final group numbers were prelimbic saline (*n* = 9), infralimbic saline (*n* = 5), prelimbic amphetamine (*n* = 7), infralimbic amphetamine (*n* = 7), prelimbic flupenthixol (*n* = 9), and infralimbic flupenthixol (*n* = 7).

### Cumulative conditioned cue and place preference in test 7

**Fig. 3:**
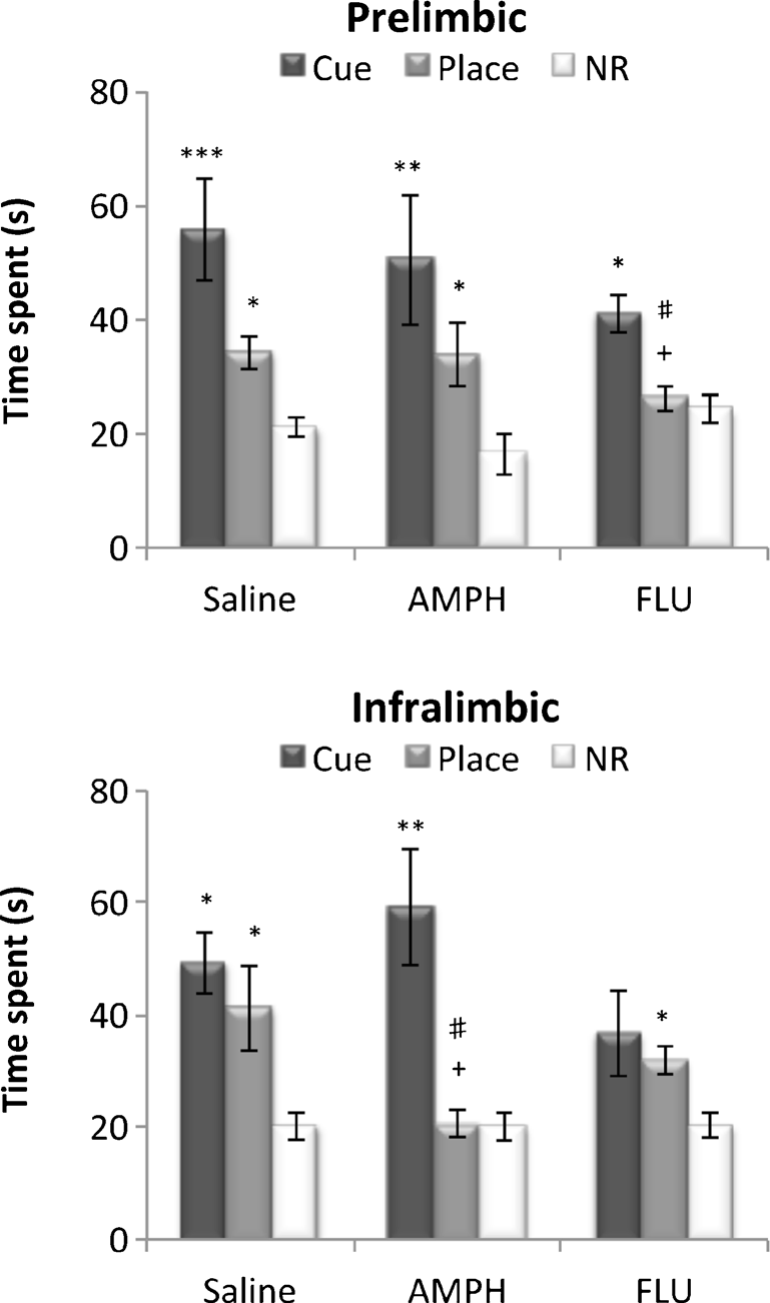
The time spent in the cue, place, and non-rewarded (*NR*) arms during conditioned cue and place preference test 7 (5 min). Performance of animal groups with saline, amphetamine (*AMPH*), and *cis*-flupenthixol (*FLU*) infused into the prelimbic cortex (*top*) and infralimbic cortex (*bottom*) is expressed as the mean ± SEM time spent in the cue arm, the place arm, and the mean of the four NR arms. *p < 0.05, **p < 0.01, ***p < 0.001, significant difference between the time spent in the cue/place arm and NR arms. +*p* < 0.05, #*p* < 0.05, significant difference between time spent in the “place arm” between drug treatment groups

An overall ANOVA on the performance of the animals in test 7 in session 8 (Fig. 3) was conducted to examine the cumulative outcome of cue and place conditioning. It was evident that the saline-infused groups showed significant preference for the cue and place arms over the non-rewarded arms in test 7.

However, repeated pre-training flupenthixol infusions into the PL cortex and repeated pre-training amphetamine infusions into the IL cortex both impaired conditioned place preference.

Conversely, repeated flupenthixol infusions into the IL attenuated conditioned cue preference. A three-way ANOVA confirmed a significant arm × drug treatment × region interaction (*F*(4, 76) = 2.68, *p* < 0.05) and significant main effects of drug treatment (*F*(2, 38) = 4.69, *p* < 0.05) and arm (*F*(2, 76) = 40.84, *p* < 0.0001).

Subsequent simple main effects analyses revealed a significant effect of drug treatment in the PL (*F*(2, 38) = 5.01, *p* < 0.02) and IL (*F*(2, 38) = 5.42, *p* < 0.01) for conditioned place preference, which was due to the time spent in the place arm being significantly reduced in the intra-PL flupenthixol infused, and intra-IL amphetamine-infused animals, compared to that in the respective saline-infused groups (*p* < 0.05). Results also showed a significant main effect of arm in all groups (prelimbic: saline, *F*(2, 37) = 10.46, *p* < 0.0001; amphetamine, *F*(2, 37) = 10.11, *p* < 0.0001; flupenthixol, *F*(2,37) = 3.66, *p* < 0.05; infralimbic: saline, *F*(2, 36) = 7.69, *p* < 0.01; amphetamine, *F*(2, 36) = 9.23, *p* < 0.01; flupenthixol, *F*(2, 36) = 4.40, *p* < 0.02). Bonferroni corrected post hoc comparisons revealed that all groups showed significant preference for the cue arm over the NR arm except for the IL flupenthixol-infused group (*p* = 0.14). Significant preference for the place arm over the NR arm was observed in all groups except for the PL flupenthixol-infused (*p* = 1.0) and IL amphetamine-infused (*p* = 0.71) groups. Finally, there was a significant simple main effect of region in the performance of conditioned place preference with both amphetamine (*F*(1, 38) = 5.19, *p* < 0.05) and flupenthixol (*F*(1, 38) = 4.93, *p* < 0.05) treatments, which was attributable to the fact that intra-IL amphetamine-infused rats spent less time in the place arm, compared to intra-PL amphetamine-infused rats (*p* < 0.05), and intra-PL flupenthixol infused rats spent less time in the place arm compared to intra-IL flupenthixol-infused rats (*p* < 0.05).

**Fig. 4:**
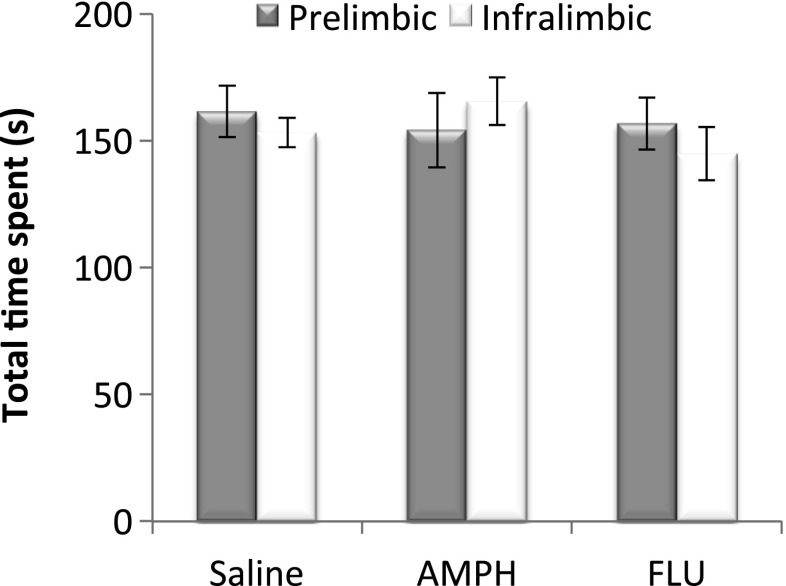
The mean ± SEM total time spent exploring the six arms of the radial maze in test 7 (5 min) in the prelimbic and infralimbic saline, amphetamine (*AMPH*), and *cis*-flupenthixol (*FLU*)-infused groups

An ANOVA on the total time spent exploring the three arms in test 7 was also conducted to assess nonspecific drug-induced effects on exploratory activity (Fig. 4). There was no significant main effect of drug (*F*(2, 38) = 0.38, *p* = 0.69), region (*F*(1, 38) = 0.11, *p* = 0.75), nor a significant interaction between drug and region (*F*(2, 38) = 0.65, *p* = 0.53) indicating that differences in exploratory activity could not have accounted for drug-induced alterations in conditioned cue and place preference.

### Within-subject pre- versus post-conditioning change in time spent in the cue and place arms

**Fig. 5:**
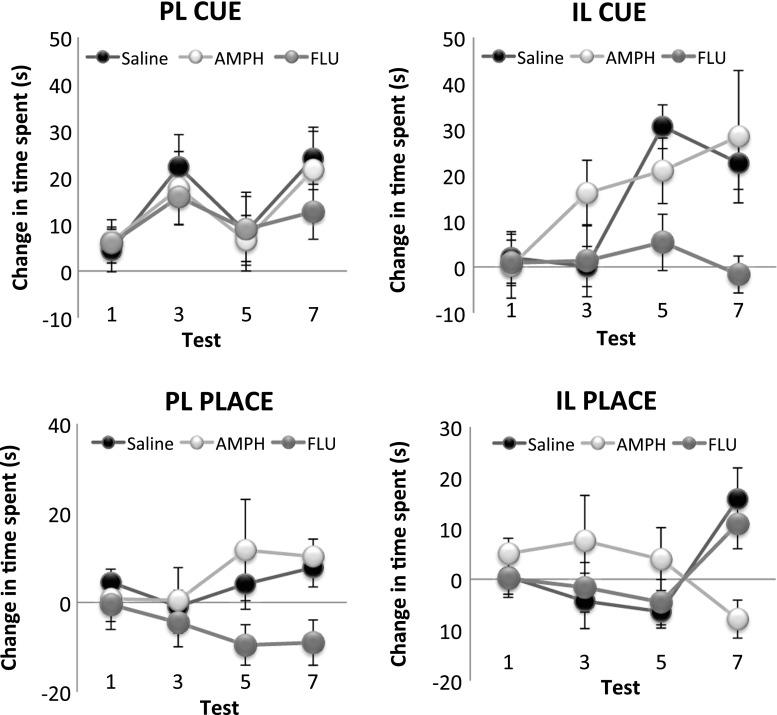
The mean ± SEM change in time spent in the cue and place arms in tests 1, 3, 5, and 7 from preconditioning baseline measures of time spent. The two *left panels* show the change in time spent in the cue (*top*) and the place (*bottom*) arms for the prelimbic (*PL*) saline, amphetamine (*AMPH*), and *cis*-flupenthixol (*FLU*) treatment groups, while the two *right panels* show the change in time spent in the cue (*top*) and the place (*bottom*) arm for the infralimbic (*IL*) saline, amphetamine (*AMPH*), and *cis*-flupenthixol (*FLU*) treatment groups

As another measure of the acquisition of conditioned cue and place preference, the change in the time spent in the cue and place arms from a preconditioning habituation (baseline) session to post-conditioning tests 1–7 was calculated for each rat (Fig. 5). This measure provides an index of cue and place learning in individual animals that is not affected by potential between-subject differences in baseline locomotor activity. An overall ANOVA revealed a significant three-way interaction between arm, drug, and region (*F*(2, 38) = 3.38, *p* < 0.05), as well as significant main effects of arm (*F*(2, 76) = 27.47, *p* < 0.0001) and drug (*F*(2, 38) = 4.00, *p* < 0.03). Subsequent simple main effects analyses revealed a significant simple main effect of drug treatment in the IL for the acquisition of conditioned cue preference (*F*(2, 38) = 4.10, *p* < 0.03) and in the PL for the acquisition of conditioned place preference (*F*(2, 38) = 4.92, *p* < 0.02). Bonferroni corrected post hoc comparisons revealed a significant difference in the change in the time spent in the place arm between the PL saline-infused and flupenthixol-infused groups (*p* < 0.05), with the former showing significantly increased time spent in the place arm and the latter showing decreased time spent in the place arm. Furthermore, there was a significant difference in the change in time spent in the cue arm between IL saline-infused and flupenthixol-infused groups (*p* < 0.05), with the saline-infused group showing increased time spent in the cue arm and the flupenthixol-infused group showing decreased time spent in the cue arm.

Taken together, analyses conducted on two different measures of cue and place learning, (1) conditioned preference measure and (2) pre- versus post-conditioning changes in the time spent in the arms across the seven tests, support the following results: Repeated pre-training flupenthixol infusions into the PL, and repeated amphetamine infusions into the IL, significantly attenuated levels of conditioned place preference. In contrast, repeated pre-training flupenthixol infusions into the iIL selectively attenuated conditioned cue preference while sparing conditioned place preference.

## Discussion

The present study provides evidence for a functional dissociation of IL and PL DA in regulating limbic control over appetitive behavior. Spatial control over appetitive learning (conditioned place preference) was selectively attenuated by repeated pre-training flupenthixol-induced blockade of dopaminergic D1/D2 receptors in the PL and by repeated amphetamine-induced augmentation of DA in the IL. In contrast, repeated blockade of DA neurotransmission in IL impaired cue conditioning (conditioned cue preference) while sparing spatial conditioning. These findings demonstrate that dopaminergic neurotransmission in the IL and PL has functionally opposite roles in regulating HPC-dependent spatial control over appetitive behavior, while IL DA may also contribute to the regulation of BLA-dependent cue learning.

### Role of PL DA in mediating spatial control over appetitive conditioning

We have previously demonstrated that cue and place conditioning is mediated differentially by the HPC and BLA, with the former being critical for conditioned place preference and the latter being critical for conditioned cue preference (Ito et al. 2008; Ito and Canseliet 2010). Furthermore, the HPC and BLA, together with the NAc shell and core subregions, form distinct limbic–striatal systems that support contextual/spatial and discrete cue control over appetitive behavior, respectively. Thus, the HPC and NAc shell form a functional pathway that supports spatial contextual control over Pavlovian reward seeking and context-induced reinstatement of drug-seeking behavior (Bossert et al. 2012; Chaudhri et al. 2010; Ito et al. 2008), while the basolateral amygdala and nucleus accumbens core form a circuit that is concerned with discrete cue control over Pavlovian reward learning and cue-induced drug-seeking behavior (Everitt et al. 1991; Fuchs et al. 2005; Ito et al. 2008). We have also previously shown that dopaminergic mechanisms within the NAc regulate the competition of HPC and BLA inputs over appetitive behavior. Repeated pre-training amphetamine microinfusions in the NAc shell selectively enhanced spatial control over appetitive learning, while repeated amphetamine administration in the NAc core had the opposite effect of attenuating spatial control over learning (Ito and Hayen 2011). In the present study, repeated pre-training blockade of dopaminergic D1/D2 receptors in the PL cortex, an area that is well connected to the NAc core and shell, led to a selective decrease in spatial (HPC dependent) control, while sparing discrete cue (BLA dependent) control over Pavlovian approach behavior, indicating the critical importance of PL dopamine in regulating HPC-dependent processes. This finding is consistent with the large body of evidence that indicates a close anatomical and functional link between the HPC and PL in spatial tasks (Condé et al. 1995; Floresco et al. 1997; Jay and Witter 1991; Seamans et al. 1995, 1998).

### Opposing roles of IL and PL DA in spatial information processing

In the present study, while a blockade of DA neurotransmission induced by repeated infusions of *cis*-flupenthixol into the PL attenuated conditioned place preference, the same effect was produced by repeated infusions of amphetamine into the IL. This suggests that DA neurotransmission in these two regions not only has functionally dissociable roles, but also *opposite* roles in the regulation of HPC-dependent learning. This is consistent with other reports of functionally opposing interactions between the IL and PL (Killcross and Coutureau 2003; Peters et al. 2008; Vidal-Gonzalez et al. 2006). For instance, Killcross and Coutureau (2003) showed that in contrast to the effects of PL lesions, which impair rats’ capacity for goal-directed actions and leave habit-based learning intact, lesions of the IL had the opposite effect of impairing the acquisition of habit-based learning. One proposed mechanism for the gradual transition from goal-directed to habit-based learning is that the IL exerts an inhibitory influence over the PL. More recently, Hitchcott et al. (2007) showed ventromedial (IL) dopamine to be important in exerting bidirectional control over instrumental responding, adjusting responses to changing outcome values. Similarly, Vidal-Gonzalez et al. (2006) found that brief microstimulation of PL increased the expression of conditioned fear and prevented extinction, while the same treatment in the IL had the opposite effect of reducing conditioned fear. Finally, Peters et al. (2008) showed that increasing activity in PL reinstated cocaine-seeking behavior, while activation of IL had the effect of suppressing reinstatement of drug-seeking behavior. Together, these findings suggest a role for IL and its dopaminergic innervation as a potential “brake” that inhibits PL-mediated influences over appetitive and aversive behavior, allowing for a more selective control of limbic information over behavior. A recent electrophysiological study also supports the notion of a hierarchical functional organization between the PL and IL in learning and memory processes, with reports of highly coherent fast network oscillations in the PL and IL that disappear when the two structures are disconnected. The oscillations in the IL were found to be more powerful than that in the PL during interactions, possibly indicating the IL to be the driving force of IL–PL interactions (Van Aerde et al. 2008).

### Role of IL DA in mediating discrete cue control over appetitive conditioning

While repeated infusions of *cis*-flupenthixol into the PL impaired conditioned place preference, infusion of *cis*-flupenthixol into the IL attenuated conditioned cue preference while sparing conditioned place preference. This finding contributes to the growing number of studies showing the functional heterogeneity of PL and IL in discrete cue and contextual control over drug- and reward-seeking behavior.

Our findings implicate IL DA neurotransmission in the processing of BLA-dependent, discrete cue-reward association, which is consistent with other studies that have reported the recruitment of IL in cue-induced reinstatement of methamphetamine seeking (Rocha and Kalivas 2010) and cocaine seeking (LaLumiere et al. 2012). The capacity of IL DA to regulate cue-induced reward associations can be understood in light of its connectivity to the amygdala. IL contributes the majority of the mPFC inputs to the amygdala (Mcdonald et al. 1996; Vertes 2004) and in turn receives reciprocal innervation from BLA (McDonald et al. 1996). These findings, together with the results from the present study, highlight the key role of IL DA in regulating information processing from the amygdala and, more specifically, potentially enhancing BLA control over appetitive behavior.

The absence of any effect of repeated amphetamine or flupenthixol infusions in the PL on conditioned cue preference is at first sight surprising, given previous findings implicating PL and its afferents to the NAc core in discrete cue-induced methamphetamine and heroin seeking (LaLumiere and Kalivas 2008; Miguéns et al. 2008; Rocha and Kalivas 2010). However, notable procedural differences could account for the observed differences; for instance, preconditioning pharmacological manipulations in this study versus post-training PL disruption employed in other studies, and the use of Pavlovian learning in this study versus instrumental learning paradigms in other studies. Clearly, the particular circumstances under which the PL and its dopaminergic mechanisms are critically involved in discrete cue control over appetitive behavior warrant further investigation.

### Functional interaction between mPFC and NAc DA  in regulating limbic control over appetitive behavior

Our previous findings showed that NAc shell dopamine augmentation selectively enhanced HPC-dependent spatial control over appetitive behavior (Ito and Hayen 2011), while our current findings suggest a role of IL DA in inhibiting spatial processing. Together, these findings suggest a possible reciprocity of function between the dopaminergic innervations of IL and the NAc shell in regulating limbic control over appetitive behavior, in accord with previous pharmacological studies showing an inverse relationship between mesoaccumbens and mesocortical dopamine responses, with the latter regulating the former via direct glutamatergic projections or indirectly through the ventral tegmental area (Karreman and Moghaddam 1996; Ventura et al. 2004). Furthermore, our findings support the hypothesis that IL activation generally regulates or inhibits the control of subcortically generated motivational information over behavior (Richard and Berridge 2012). Indeed, Richard and Berridge (2012) found that intense motivations such as eating and fear-related behaviours generated by NAc shell glutamate disruptions were powerfully inhibited by the activation of IL. LaLumiere et al. (2012) further showed that IL and NAc shell interact bidirectionally in regulating cocaine-seeking behavior, in that the activation of IL inhibited reinstatement, while activation of DA neurons in the NAc shell promoted cocaine-seeking behavior after extinction. Such a role is conceivable, given that IL projects selectively to the NAc shell (Berendse et al. 1992; Vertes 2004) and may interact antagonistically to dampen NAc shell influence over behavior. However, this opposing function of IL and NAc shell appears to be drug specific, as Bossert et al. (2012) recently showed.

It must be noted, however, that while the primary action of amphetamine is upon the dopamine system, there is a possibility that alterations in other monoaminergic systems may have contributed to the attenuating effects of IL amphetamine microinfusions upon conditioned place preference. There is evidence, for instance, that prefrontal norepinephrine neurotransmission is involved in the attribution of motivational salience to stimuli (as assessed by conditioned place preference) by enhancing dopaminergic transmission in the NAc, while prefrontal dopamine has the opposite effect of inhibiting dopamine neurotransmission in the NAc (Ventura et al. 2003). However, our finding of a functionally opposite outcome of amphetamine microinfusions into the IL and NAc shell in the modulation of HPC-dependent learning can be better explained by the effects of amphetamine upon mesoaccumbens and mesocortical dopamine systems. Thus, amphetamine-induced augmentation in dopamine levels would have led to an inhibition of dopamine neurotransmission in the NAc shell, thereby attenuating HPC-dependent learning. Nevertheless, further investigation would need to be conducted to assess the effects of selectively targeting the prefrontal noradrenaline system in the modulation of limbic control over appetitive behavior.

In summary, the present findings suggest that DA neurotransmission in the PL and IL has distinct, and even opposing, roles in the regulation of limbic information processing in the medial prefrontal cortex. PL DA is critical for allowing HPC inputs to gain control over appetitive behavior, whereas IL DA has a twofold role, not only in allowing preferential processing of BLA inputs, but also to dampen HPC-PL-mediated information. This is consistent with the idea that IL may perform a regulatory function, exerting stringent control over motivations or emotions gaining influence over behavior.

## References

[CR1] Berendse HW, Graaf YG, Groenewegen HJ (1992). Topographical organization and relationship with ventral striatal compartments of prefrontal corticostriatal projections in the rat. J Comp Neurol.

[CR2] Bossert JM, Stern AL, Theberge FRM, Marchant NJ, Wang HL, Morales M, Shaham Y (2012) Role of projections from ventral medial prefrontal cortex to nucleus accumbens shell in context-induced reinstatement of heroin seeking. J Neurosci 32:4982–4991 10.1523/JNEUROSCI.0005-12.2012 PMC3335169 22492053

[CR3] Chaudhri N, Sahuque LL, Schairer WW, Janak PH (2010). Separable roles of the nucleus accumbens core and shell in context- and cue-induced alcohol-seeking. Neuropsychopharmacology.

[CR4] Chiba T, Kayahara T, Nakano K (2001). Efferent projections of infralimbic and prelimbic areas of the medial prefrontal cortex in the Japanese monkey, *Macaca fuscata*. Brain Res.

[CR5] Condé F, Maire-Lepoivre E, Audinat E, Crépel F (1995). Afferent connections of the medial frontal cortex of the rat. II. Cortical and subcortical afferents. J Comp Neurol.

[CR6] Coutureau E, Killcross S (2003). Inactivation of the infralimbic prefrontal cortex reinstates goal-directed responding in overtrained rats. Behav Brain Res.

[CR7] Euston DR, Gruber AJ, McNaughton BL (2012). The role of medial prefrontal cortex in memory and decision making. Neuron.

[CR8] Everitt BJ, Robbins TW (2005). Neural systems of reinforcement for drug addiction: from actions to habits to compulsion. Nat Neurosciene.

[CR9] Everitt BJ, Morris KA, O’Brien A, Robbins TW (1991). The basolateral amygdala-ventral striatal system and conditioned place preference: further evidence of limbic-striatal interactions underlying reward-related processes. Neuroscience.

[CR10] Faure A, Haberland U, Condé F, Massioui NE (2005) Lesion to the nigrostriatal dopamine system disrupts stimulus-respones habit formation. J Neurosci 25:2771–2780 10.1523/JNEUROSCI.3894-04.2005 PMC6725127 15772337

[CR11] Floresco SB, Seamans JK, Phillips AG (1997). Selective roles for hippocampal, prefrontal cortical, and ventral striatal circuits in radial-arm maze tasks with or without a delay. J Neurosci.

[CR12] Fuchs RA, Evans KA, Ledford CC, Parker MP, Case JM, Mehta RH, See RE (2005). The role of the dorsomedial prefrontal cortex, basolateral amygdala, and dorsal hippocampus in contextual reinstatement of cocaine seeking in rats. Neuropsychopharmacology.

[CR13] Hitchcott PK, Quinn JJ, Taylor JR (2007). Bidirectional modulation of goal-directed actions by prefrontal cortical dopamine. Cereb Cortex.

[CR14] Hoover WB, Vertes RP (2007). Anatomical analysis of afferent projections to the medial prefrontal cortex in the rat. Brain Struct Funct.

[CR15] Ishikawa A, Nakamura S (2003). Convergence and interaction of HPC and amygdala projection within the PFC in the rat.. J Neurosci.

[CR16] Ito R, Canseliet M (2010). Amphetamine exposure selectively enhances hippocampus-dependent spatial learning and attenuates amygdala-dependent cue learning. Neuropsychopharmacology.

[CR17] Ito R, Hayen A (2011). Opposing roles of nucleus accumbens core and shell dopamine in the modulation of limbic information processing. J Neurosci.

[CR18] Ito R, Robbins TW, Pennartz CM, Everitt BJ (2008). Functional interaction between the hippocampus and nucleus accumbens shell is necessary for the acquisition of appetitive spatial context conditioning. J Neurosci.

[CR19] Jay TM, Witter MP (1991). Distribution of hippocampal CA1 and subicular efferents in the prefrontal cortex of the rat studied by means of anterograde transport of *Phaseolus vulgaris*-leucoagglutinin. J Comp Neurol.

[CR20] Karreman M, Moghaddam B (1996) The prefrontal cortex regulates the basal release of dopamine in the limbic striatum: an effect mediated by ventral tegmental area. J Neurochem 66:589–598 10.1046/j.1471-4159.1996.66020589.x 8592128

[CR21] Killcross S, Coutureau E (2003). Coordination of actions and habits in the medial prefrontal cortex of rats. Cereb Cortex.

[CR22] Lacroix L, Broersen LM, Feldon J, Weiner I (2000). Effects of local infusions of dopaminergic drugs into the medial prefrontal cortex of rats on latent inhibition, prepulse inhibition and amphetamine induced activity. Behav Brain Res.

[CR23] LaLumiere RT, Kalivas PW (2008). Glutamate release in the nucleus accumbens core is necessary for heroin seeking. J Neurosci.

[CR24] LaLumiere RT, Smith KC, Kalivas PW (2012). Neural circuit competition in cocaine-seeking: roles of the infralimbic cortex and nucleus accumbens shell. Eur J Neurosci.

[CR25] Mcdonald AJ, Mascagni F, Guo L (1996). Projections of the medial and lateral prefrontal cortices to the amygdala: a *Phaseolus vulgaris* leucoagglutinin study in the rat. Neuroscience.

[CR26] Miguéns M, Crespo JA, Olmo N, Higuera-Matas A, Montoya GL, García-Lecumberri C, Ambrosio E (2008). Differential cocaine-induced modulation of glutamate and dopamine transporters after contingent and non-contingent administration. Neuropharmacology.

[CR27] Naneix F, Marchand AR, Scala G, Pape J, Coutureau E, Naneix F, Marchand AR, Scala GD, Pape J, Coutureau E (2009). A role for medial prefrontal dopaminergic innervation in instrumental conditioning. J Neurosci.

[CR28] Nelson A, Killcross S (2006). Amphetamine exposure enhances habit formation. J Neurosci.

[CR29] Nordquist RE, Voorn P, de Mooij-van Malsen JG, Joosten RN, Pennartz CM, Vanderschuren LJ (2007). Augmented reinforcer value and accelerated habit formation after repeated amphetamine treatment. Eur Neuropsychopharmacology.

[CR30] Peters J, LaLumiere RT, Kalivas PW (2008). Infralimbic prefrontal cortex is responsible for inhibiting cocaine seeking in extinguished rats. J Neurosci.

[CR31] Pezze MA, Bast T, Feldon J (2003). Significance of dopamine transmission in the rat medial prefrontal cortex for conditioned fear. Cereb Cortex.

[CR32] Quirk GJ, Mueller D (2008). Neural mechanisms of extinction learning and retrieval. Neuropsychopharmacology.

[CR33] Richard JM, Berridge KC (2012). Prefrontal cortex modulates desire and dread generated by nucleus accumbens glutamate disruption. Biol Psychiatry.

[CR34] Rocha A, Kalivas PW (2010). Role of the prefrontal cortex and nucleus accumbens in reinstating methamphetamine seeking. Eur J Neurosci.

[CR35] Seamans JK, Floresco SB, Phillips AG (1995). Functional differences between the prelimbic and anterior cingulate regions of the rat prefrontal cortex. Behav Neurosci.

[CR36] Seamans JK, Floresco SB, Phillips AG (1998). D1 receptor modulation of hippocampal-prefrontal cortical circuits integrating spatial memory with executive functions in the rat. J Neurosci.

[CR37] Van Aerde KI, Heistek TS, Mansvelder HD (2008). Prelimbic and infralimbic prefrontal cortex interact during fast network oscillations. PLoS One.

[CR38] Ventura R, Alcaro A, Cabib S, Conversi D, Mandolesi L, Puglisi-Allegra S (2004). Dopamine in the medial prefrontal cortex controls genotype-dependent effects of amphetamine on mesoaccumbens dopamine release and locomotion. Neuropsychopharmacology.

[CR39] Ventura R, Cabib S, Alcaro A, Orsini C, Puglisi-Allegra S (2003). Norepinephrine in the prefrontal cortex is critical for amphetamine-induced reward and mesoaccumbens dopamine release. J Neurosci.

[CR40] Vertes RP (2004). Differential projections of the infralimbic and prelimbic cortex in the rat. Synapse.

[CR41] Vidal-Gonzalez I, Vidal-Gonzalez B, Rauch SL, Quirk GJ (2006). Microstimulation reveals opposing influences of prelimbic and infralimbic cortex on the expression of conditioned fear. Learn & Mem.

